# Systematic comparison of fluorescence imaging in the near-infrared and shortwave-infrared spectral range using clinical tumor samples containing cetuximab-IRDye800CW

**DOI:** 10.1117/1.JBO.30.S1.S13708

**Published:** 2024-11-15

**Authors:** Bas Keizers, Thomas S. Nijboer, Christa A. M. van der Fels, Marius C. van den Heuvel, Gooitzen M. van Dam, Schelto Kruijff, Igle Jan de Jong, Max J. H. Witjes, Floris J. Voskuil, Dimitris Gorpas, Wesley R. Browne, Pieter J. van der Zaag

**Affiliations:** a University Medical Centre Groningen, Department of Nuclear Medicine and Molecular Imaging, Groningen, The Netherlands; b University Medical Centre Groningen, Optical Molecular Imaging Groningen, Groningen, The Netherlands; c University Medical Centre Groningen, Department of Surgery, Groningen, The Netherlands; d University Medical Centre Groningen, Department of Oral and Maxillofacial Surgery, Groningen, The Netherlands; e University Medical Centre Groningen, Department of Urology, Groningen, The Netherlands; f University Medical Centre Groningen, Department of Pathology and Medical Biology, Groningen, The Netherlands; g AxelaRx/TRACER B.V., Groningen, The Netherlands; h Helmholtz Zentrum München, Institute of Biological and Medical Imaging, Neuherberg, Germany; i Technische Universität München, School of Medicine and Health, Chair of Biological Imaging at the Central Institute for Translational Cancer Research (TranslaTUM), München, Germany; j University of Groningen, Stratingh Institute for Chemistry, Faculty of Science and Engineering, Groningen, The Netherlands; k University of Groningen, Zernike Institute, Molecular Biophysics, Groningen, The Netherlands

**Keywords:** optical imaging, fluorescence-guided surgery, fluorescence molecular imaging, near-infrared imaging, shortwave-infrared imaging, contrast-to-noise ratio

## Abstract

**Significance:**

Shortwave-infrared (SWIR) imaging is reported to yield better contrast in fluorescence-guided surgery than near-infrared (NIR) imaging, due to a reduction in scattering. This benefit of SWIR was shown in animal studies, however not yet in clinical studies with patient samples.

**Aim:**

We investigate the potential benefit of SWIR to NIR imaging in clinical samples containing cetuximab-IRDye800CW in fluorescence-guided surgery.

**Approach:**

The potential of the epidermal growth factor-targeted NIR dye cetuximab-IRDye800CW in the shortwave range was examined by recording the absorption and emission spectrum. An *ex vivo* comparison of NIR and SWIR images using clinical tumor samples of patients with penile squamous cell carcinoma (PSCC) and head and neck squamous cell carcinoma (HNSCC) containing cetuximab-IRDye800CW was performed. The comparison was based on the tumor-to-background ratio and an adapted contrast-to-noise ratio (aCNR) using the standard of care pathology tissue assessment as the golden standard.

**Results:**

Based on the emission spectrum, cetuximab-IRDye800CW can be detected in the SWIR range. In clinical PSCC samples, overall SWIR imaging was found to perform similarly to NIR imaging (NIR imaging is better than SWIR in the 2/7 criteria examined, and SWIR is better than NIR in the 3/7 criteria). However, when inspecting HNSCC data, NIR is better than SWIR in nearly all (5/7) examined criteria. This difference seems to originate from background autofluorescence overwhelming the off-peak SWIR fluorescence signal in HNSCC tissue.

**Conclusion:**

SWIR imaging using the targeted tracer cetuximab-IRDye800CW currently does not provide additional benefit over NIR imaging in *ex vivo* clinical samples. Background fluorescence in the SWIR region, resulting in a higher background signal, limits SWIR imaging in HNSCC samples. However, SWIR shows potential in increasing the contrast of tumor borders in PSCC samples, as shown by a higher aCNR over a line.

## 1 Introduction

Treatment for most solid cancer types predominantly consists of radical surgical resection of all tumor tissue. Differentiating normal from tumor tissue intraoperatively, let alone detecting microscopic residual disease, remains challenging, even for an experienced surgical oncologist. Therefore, it is not rare that a tumor-positive margin is reported during pathology assessment 2 to 5 days after surgery.^1^^–^^3^ Literature rates of tumor-positive margins (TPM) range from 10 to 35%, depending on tumor type.^4^ If tumor tissue is present at, or near the border of the resected tissue, the risk of local recurrence and distant metastasis is increased, resulting in a decreased disease-free survival (DFS) and overall survival (OS).^5^^–^^9^ Consequently, a TPM necessitates additional treatment, such as re-operation, radiation therapy, and/or systemic therapy, which is associated with increased morbidity and a higher psychological burden on the patient.^4^^,^^6^^,^^10^^–^^12^ Accurate visualization of tumor tissue during oncological surgery is crucial and could prevent additional therapy. However, currently available intraoperative imaging techniques and both visual and tactile information obtained by the surgeon, are insufficient to adequately determine tumor margins. Thus, new techniques that provide real-time tumor visualization are sought, aiming to prevent TPM and avoid additional treatment and morbidity, hopefully increasing OS and DFS for future patients.^13^^–^^17^

Fluorescence molecular imaging (FMI) is one such technique gaining interest because of the possibility for real-time tumor visualization^17^^–^^19^ deployed both during surgical treatment of the patient (*in vivo*) and immediately after excision of the specimen (*ex vivo*).^1^^–^^3^^,^^20^^–^^23^ FMI is versatile in its clinical use; untargeted fluorescent dyes such as indocyanine green (ICG) are used for imaging tissue perfusion, while targeted fluorescent dyes are used for imaging tumor tissue and infection and track medicinal therapy by targeting cellular receptors.^13^^,^^24^^–^^26^ Even though research on FMI in the near-infrared (NIR) spectral range (700 to 900 nm) has shown promising results over the past decades, leading to 20 FMI systems^27^ and three targeted tracers^28^^–^^30^ receiving approvals by the US Food and Drug Administration (FDA), some drawbacks still remain. Scattering of light and absorbance by biological components, such as water and blood, contribute to attenuation of the excitation light, reducing the sensitivity and contrast of fluorescence images.^31^ Besides this, with respect to the short-wave infrared (SWIR) window, the degree of autofluorescence in the NIR spectral range and limited tissue penetration are some of the additional issues encountered in imaging at this spectral range.^32^^,^^33^

Currently, new techniques to enhance sensitivity, contrast, and tissue penetration are explored (e.g., multispectral optoacoustic tomography^34^ and FMI using the SWIR spectral range (1000 to 1700 nm)^35^). Preclinical studies of SWIR FMI have yielded images with enhanced contrast compared with the NIR range, because of significantly reduced autofluorescence^36^ and sharply reduced scattering (i.e., as scattering is proportional to $\lambda^{-4}$ where λ is the wavelength).^35^^,^^37^^,^^38^ Deeper tissue penetration is also a benefit of SWIR imaging. For example, skin penetration for NIR, especially in the range of current fluorescent dyes (750 to 800 nm) was estimated to be ∼2.2  mm, whereas a maximum skin penetration of 3.5 mm was found at 1090 nm.^39^

Nevertheless, SWIR FMI has not been investigated until the last decade because conventional silicon NIR detectors lack sensitivity in the SWIR region and because of previous poor commercial availability of the SWIR Indium Gallium Arsenide (InGaAs) detectors. Nowadays, as InGaAs detectors have become more readily available, a significant number of animal studies investigating SWIR FMI have been performed showing deeper tissue penetration, higher contrast, and increased tumor-to-background ratio (TBR).^31^^,^^40^^–^^42^ Clinical studies, however, are still lacking, probably due to the scarcity of efficient and clinically available SWIR dyes. Development of such SWIR dyes is ongoing^43^ and may improve SWIR performance. Yet, these dyes still require regulatory approval before they can be applied clinically. Interestingly, recent studies have shown that the emission tails of the NIR dyes such as ICG and IRDye800CW appear to extend far in the SWIR spectral range and outperform current available SWIR dyes in terms of quantum yield.^44^^–^^46^ Because these NIR dyes are available and approved for (investigational) clinical use, this opens the possibility for fast translation of SWIR FMI into clinical application.

Here, we report the investigation of the potential clinical benefit of SWIR imaging of tumor tissue in surgical oncology compared with NIR imaging. For this, SWIR imaging was compared with imaging in the NIR spectral range using fluorescence images of clinical samples. To perform this comparison, the optical properties of ICG and IRDye800CW in the SWIR spectral range have been evaluated and the optimal NIR imaging system currently available was selected. Thereby enabling a systematic comparison between SWIR and NIR spectral imaging in tumor visualization using the epidermal growth factor receptor (EGFR)-targeted NIR dye, cetuximab-IRDye800CW, in clinical samples of penile squamous cell carcinoma (PSCC) and head and neck squamous cell carcinoma (HNSCC). All measurements were performed *ex vivo*, as assessment of excised specimens, outside the sterile working area, is easier to standardize and is less susceptible to regulatory issues, enabling faster translation into clinical practice.^17^ To the best of our knowledge, this is the first time the SWIR and NIR imaging results are compared using clinical samples containing clinically relevant and optimal doses of this targeted dye.^2^ Improving FMI methods could lead to improved tumor margin assessment, benefitting patient outcomes.

## 2 Methods

This research was divided into three stages to address the research question comprehensively: (A) the potential of IRDye800CW in the SWIR spectral range was investigated and compared with the untargeted dye ICG, in which fluorescence imaging in the SWIR spectral range is already proven to be beneficial;^45^ (B) the optimal NIR imaging system was selected for comparison to the SWIR imaging system; and (C) the SWIR imaging system was optimized and a systematic comparison between NIR and SWIR imaging using clinical samples was made. The three stages are discussed accordingly.

### 2.1 Potential of IRDye800CW in the SWIR Range

The potential application of IRDye800CW in SWIR fluorescence imaging was investigated by measuring the infrared spectrum of the targeted dye cetuximab-IRDye800CW at various concentrations and by comparing the emission spectrum to the spectrum of ICG, a NIR dye that was more frequently used for SWIR imaging in earlier studies.^31^^,^^41^^,^^45^

Briefly, cetuximab-IRDye800CW (molar weight, MW 148,114  g/mol) was produced using commercially available cetuximab (Erbitux^®^) 5  mg/ml and NIR fluorescence dye IRDye800CW (Li-COR Biosciences, Lincoln, NE, United States). They were conjugated and purified using PD-10 buffer exchange columns (GE Healthcare, Chicago, IL, United States). Cetuximab-IRDye800CW was formulated in a sodium-phosphate buffer at a concentration of 1  mg/ml. The exact production process has been described previously.^47^ Cetuximab-IRDye800CW was prepared at a range of concentrations using demineralized water as solvent. Final concentrations ranged from 6.8  μM down to 0.1 nM. ICG (Diagnostic Green GmbH, Aschheim, Germany, MW 775  g/mol) powder for injection was dissolved in demineralized water and was prepared at concentrations ranging from 1.3 mM to 17 nM. All samples were placed in glass cuvettes (Hellma OS 1 cm pathlength cuvettes), and the absorption spectra were recorded on a UV/vis spectrometer (Specord210 - AnalytikJena). Subsequently, emission spectra between 750 and 1000 nm were recorded with an Avantes EVO-ULS2048 complementary metal-oxide-semiconductor (CMOS) spectrometer and a 700-nm longpass filter (Thorlabs FELH0700, Newton, United States) at 660 nm excitation (Thorlabs M660FP1). The 660-nm excitation was chosen to enable the recording of the emission spectrum starting at 750 nm. Emission spectra between 1000 and 1600 nm were recorded using an InGaAs diode array detector (Andor Technology iDus-InGaAs 492-1.7, Belfast, United Kingdom) with excitation at 785 nm (diode laser, ONDAX LM-785, 75 mW at source). The laser power output was attenuated to 6 and 220  μW using a graded neutral density filter (Thorlabs NDL-25C-4). A dichroic filter (Semrock Di02-R785, Rochester, United States) was used to direct the excitation light toward the sample, and emission was collected in 180 deg backscattering arrangement with a 2.5-cm diameter/30 mm focal length planoconvex lens and passed through an 808-nm long pass (LP) filter to reject Rayleigh scattering and focused into the spectrometer (Andor Technology, iKymera-193 with a 600 l/mm grating blazed at 830 nm). An LP dichroic mirror with an 805-nm cut-on at 45 deg (Thorlabs DMLP805) was used to collect the SWIR spectrum of the dyes due to interference fringes in that region with the Di02-R785 filter.

Correction for a spectral response was performed using a calibrated halogen light source (Avantes-HAL-Cal). Correction for intensity was performed by fitting the halogen lamp emission spectral file with the black body emission spectrum, which generated a correction spectrum when combined with the recorded emission spectrum of the calibrated light source.

The concentration of cetuximab-IRDye800CW with the highest fluorescence intensity (6.8  μM) was subsequently compared with the 6.7  μM concentration of ICG.

### 2.2 NIR Imaging System Comparison

A comparison of fluorescence imaging systems based on technical specifications and fluorophore-containing phantoms has been described previously.^48^ Here, the four key NIR imaging systems approved for clinical use at our center were compared based on technical specifications and phantom measurement performance. The first imaging system was a small animal imaging system modified for clinical specimen imaging, i.e., the PEARL trilogy (Li-COR Biosciences, Lincoln, NE, United States). The other systems were intraoperative systems, i.e., Quest Spectrum (Quest Medical Imaging B.V., Middenmeer, The Netherlands), Stryker Spy Elite (Stryker, Kalamazoo, MI, United States), and SurgVision Explorer Air II (SurgVision GmbH, Munich, Germany). Eventually, the optimal NIR imaging system was compared with the Kaer Labs NIR-II system (Kaer Labs, Nantes, France).

#### 2.2.1 Technical specifications and sensitivity assessment

**Fig. 1:**
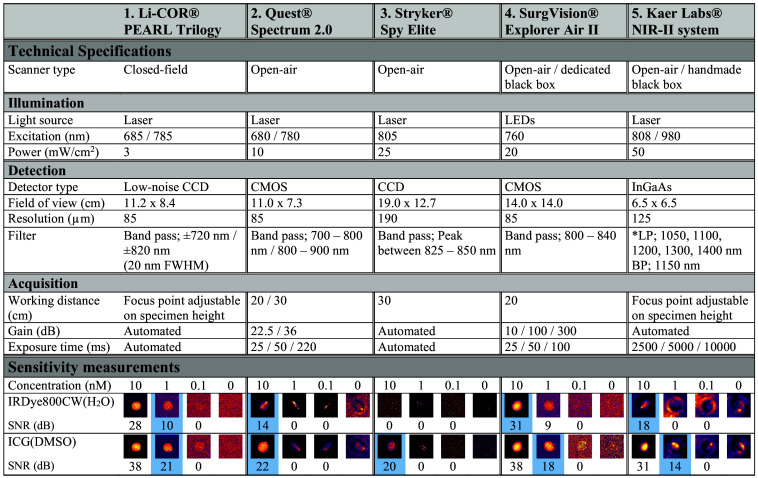
Technical specifications of the NIR and the SWIR imaging systems investigated. The lower part gives the sensitivity of each system through liquid phantom comparisons. The blue frame indicates the lowest detectable concentration of dye for each system. Abbreviations: full width at half maximum (FWHM), dimethyl sulfoxide (DMSO), long pass (LP), bandpass (BP), and signal-to-noise ratio (SNR).

Technical specifications of all imaging systems were either requested from the corresponding manufacturers or taken from product information. The investigated variables were categorized into illumination, detection, and acquisition variables (Fig. 1). Field of view and laser power were measured with a ruler and optical power meter (Thorlabs, PM100A) with a standard 50 mW photodiode power sensor (Thorlabs, S120C), respectively.

The sensitivity of the imaging systems was determined using liquid phantoms. Samples based on IRDye800CW and ICG dissolved in water ($H_{2}O$) and dimethylsulfoxide (DMSO), respectively, were prepared at concentrations of 10  μM, 1  μM, 100 nM, 10 nM, 1 nM, and 100 pM. Concentrations of both dyes were placed in microtubes (Sarstedt, colorless microtube 1.5 ml, code 72.692, screw cap). The tubes, divided into high (10  μM, 1  μM, 100 nM, and 0) and low (10 nM, 1 nM, 100 pM, and 0) concentrations, were placed in holders each holding four samples.

The liquid phantoms were imaged in a dark room using all five imaging systems. Depending on the adjustable settings, one or more fluorescence images were taken; a combination of all available settings was used, and detailed acquisition settings per system are listed in Fig. 1.

Obtained fluorescence images were analyzed using ImageJ (Fiji, version 1.53t). When available, a background image was subtracted from corresponding fluorescence images. The images were cropped to the desired region of interest (ROI) of the phantoms and were scaled between the minimum and maximum image pixel intensity. To enhance visibility the colormap “mpl-inferno” of ImageJ was used to display the images in Fig. 1. Intensity plots over a line were analyzed to determine the lowest detectable concentration. In case of a suspected reflection, also the intensity line perpendicular to this suspected reflection was analyzed. If an increased signal was seen over the entire area of a particular concentration phantom, the concentration was considered to be detectable. If only the reflection showed an increased signal, the concentration was considered to be undetectable. Finally, to prevent bias for the method described above, the signal-to-noise ratio (SNR) in decibel (dB) was calculated using the mean fluorescence intensity (MFI) over the area of the concentration phantom (reflection excluded), the MFI of the control (0 nM concentration, reflection excluded), and the standard deviation of this control:^49^

$$\mathrm{SNR}(\mathrm{dB})=20\cdot \log(\frac{{\mathrm{MFI}}_{\text{signal}}-{\mathrm{MFI}}_{\text{control}}}{\sigma_{\text{control}}}).$$ (1)

As published earlier and according to the American Association of Physicists in Medicine (AAPM) Task Group Report on the evaluation of the performance of fluorescence systems,^50^ the signal of a detectable concentration should be greater than at least 3× the standard deviation of the background, resulting in an SNR (dB) of >9.5  dB. Note that the performance of NIR and SWIR imaging systems in a clinical setting does not only depend on its sensitivity alone but also on scattering and requires evaluation using clinical samples, as discussed earlier by Koch et al.^51^ Therefore, additional measurements have been performed here.

#### 2.2.2 Solid phantom comparison

A composite solid phantom was used for the characterization of multiple imaging system parameters. This phantom is already extensively described and tested on different imaging systems.^52^ In short, using the signal intensity from the wells designated to assess the illumination profile, correction of acquired data was performed, so that systems of markedly different specifications produce the same readouts for a similar field of view. After correction for illumination spatial distribution, various compounds were segmented and quantified, resulting in an individual benchmarking score of specific properties and an overall benchmarking score to compare imaging systems, adopting the methodology described previously.^52^

As the phantom is designed for NIR imaging systems, the SWIR imaging system was excluded from this analysis. The phantom was imaged using the four NIR imaging systems. For phantom imaging with the PEARL Trilogy, Quest Spectrum, and Stryker Spy Elite a combination of all available settings was used, as mentioned earlier and listed in Fig. 1. For this phantom comparison the SurgVision Explorer Air was used instead of the SurgVision Explorer Air II due to availability. Besides differences in detector type [charged-coupled device (CCD) vs. CMOS], the technical specifications of these imaging systems are similar. The adjustable settings for the SurgVision Explorer Air were similar to the settings of the SurgVision Explorer Air II (Fig. 1). All data processing was implemented in MATLAB (Mathworks, Natick, MA, United States).

### 2.3 Comparison of Optimized NIR and SWIR Imaging Using Clinical Tumor Samples Containing Cetuximab-IRDye800CW

#### 2.3.1 Choosing optimal SWIR fluorescence imaging settings

Six interchangeable filters (Thorlabs, Newton, NJ, United States) were available for the SWIR fluorescence imaging system: LP 1050 nm, LP 1100 nm, LP 1200 nm, LP 1300 nm, LP 1400 nm, and bandpass (BP) 1150  nm±50  nm. Determination of the optimal SWIR system filter settings was performed by imaging a transparent tube (inner diameter 0.6 mm, outer diameter 1.2 mm) filled with cetuximab-IRDye800CW at a concentration of 3.4  μM. One tube was embedded in the intralipid, and another tube was covered with various layers of fresh bovine tissue at depths of 0, 1, 3, and 5 mm. Fluorescence images were obtained using the maximum exposure time without saturation of fluorescence signals, with a maximum of 10 s. A mean fluorescence signal perpendicular to the tube was acquired for all images and normalized to its own maximum. The full width at half maximum (FWHM) was obtained and plotted for all filters and depths. Finally, the obtained data were compared to the results of the NIR system to select the optimal SWIR system filter settings. All data processing was implemented in MATLAB.

#### 2.3.2 Clinical samples and compliance with ethical standards

NIR and SWIR imaging systems were compared, after the selection of the optimal system and settings, using data from clinical trials investigating the feasibility of using cetuximab-IRDye800CW for resection margin assessment in PSCC and HNSCC surgery. Both studies are approved by the Institutional Review Board of the University Medical Centre Groningen (METc 2020/300 and METc 2022/456, respectively) and conducted according to the Dutch Act on Medical Research involving Human Subject (WMO) and principles of the Declaration of Helsinki (adapted version Fortaleza, Brazil, 2013). The trials are registered at the National Institute of Health’s (NIH) National Library of Medicine dedicated website (under NCT05376202 and NCT05499065, respectively).^53^^,^^54^ Informed consent was obtained from all patients prior to any study-related procedure. Clinical grade cetuximab-IRDye800CW was produced as described in Sec. 2.1. Two days prior to surgery, the tracer was administered to each patient using the optimal dosing protocol of 75 mg unlabeled cetuximab, followed by 15 mg of cetuximab-IRDye800CW.^2^ This dosing scheme leads to the optimal fluorescence signal to discriminate between tumor and background.^2^

#### 2.3.3 Postoperative tissue processing

After excision of the tumor, the fully excised specimen was imaged sequentially using the selected NIR and SWIR imaging systems under a combination of all available exposure times and/or filters. After formalin fixation, the tissue was cut into 3- to 5-mm thick slices, which were then imaged using both camera systems and available settings. Subsequently, the tissue slices were paraffin-embedded and processed for standard histopathological examination, i.e., sectioning in 4  μm tissue sections and hematoxylin and eosin (H&E) staining.

On H&E-stained tissue slices, a board-certified pathologist, ignorant of the fluorescence data, marked tumor ROIs. Each H&E image and accompanying fluorescence images of both systems were manually overlaid to acquire tumor and healthy (background) tissue ROIs on the fluorescence images.

#### 2.3.4 Comparing NIR and SWIR fluorescence images

NIR and SWIR were compared based on images of both *ex vivo* whole tissue specimens as well as tissue slices. First, *ex vivo* whole tissue specimen fluorescence images were compared because these are more representative of the envisioned clinical use of both systems. The images were compared visually using white light images of the tumor tissue as a reference. Locations of areas with high and low fluorescence intensity were compared between NIR and SWIR images.

**Fig. 2:**
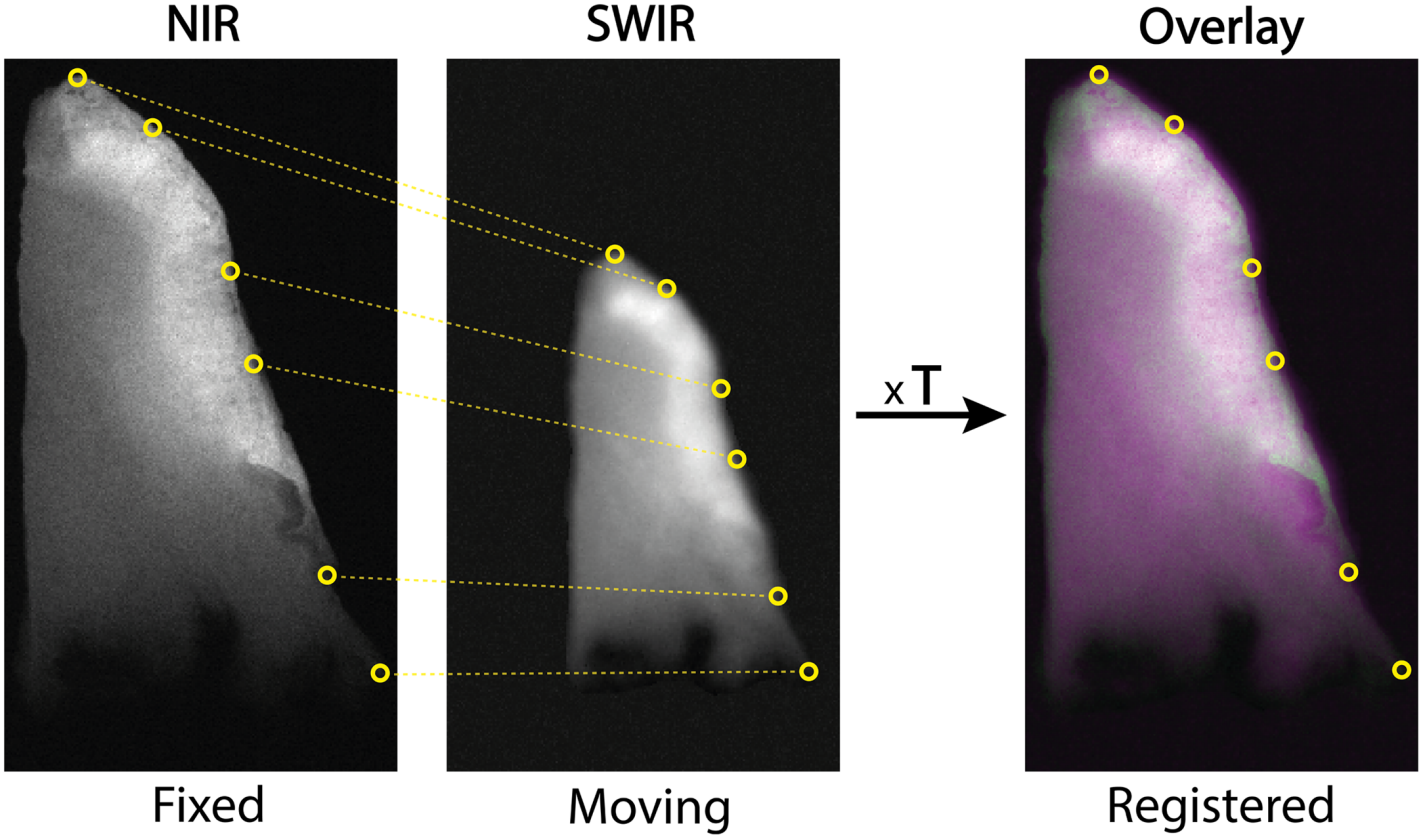
Registration of NIR and SWIR images. Several correlating points are chosen to overlay the images using a translation matrix (T).

Subsequently, the previously acquired tissue slices were used as ground truth to compare NIR and SWIR imaging more precisely. Tumor and background segmentation of these tissue slices can be obtained with great certainty as the pathologist can designate tumor tissue on H&E-stained sections, which can be correlated to the fluorescence images of corresponding tissue slices. A useful head-to-head comparison necessitates analyzing identical regions in the NIR and SWIR fluorescence images of the tissue slice. Therefore, a semi-automatic image registration was performed in MATLAB, using the “cpselect” and the “fitgeotrans” functions with similarity transformation type to acquire a transformation matrix (T) (Fig. 2).^55^^,^^56^

Following image registration, various analysis methods were used to compare NIR and SWIR imaging. First, based on the acquired ROIs using H&E staining, a tumor and background MFI and standard deviation (σ) were calculated. Next, the TBR and contrast-to-noise ratio (CNR) were calculated for the whole tissue slice. The TBR was calculated over the whole tissue slice as this is the current standard of fluorescence image assessment.^57^ It is calculated using the ratio of the MFI of the tumor and the MFI of the background

$$\mathrm{TBR}=\frac{{\mathrm{MFI}}_{\text{tumor}}}{{\mathrm{MFI}}_{\text{background}}}.$$ (2)

The CNR is a relatively new measure compared with TBR and is recommended in recent literature as it is more informative on the detectability of contrast.^58^ CNR is calculated using the MFI of the tumor and background and the σ of the background. The CNR in this study was adapted with respect to the standard definition to an adapted contrast-to-noise ratio (aCNR), as the standard deviation of the background is scaled by a factor of two, which should make the formula more robust to noise

$$\mathrm{aCNR}=\frac{{\mathrm{MFI}}_{\text{tumor}}-{\mathrm{MFI}}_{\text{background}}}{2\times \sigma_{\text{background}}}.$$ (3)

Next, four individual lines per tissue slice were manually drawn through tumor tissue, perpendicular to the tumor edge as indicated by the pathologist. Each individual line was widened to obtain a sub-image of 11 pixels wide. Subsequently, to reduce noise a filtered line of one pixel wide was acquired by taking a Gaussian mean of the 11-pixel wide sub-image. As the tumor boundary is known based on the H&E results of pathology, each pixel on this filtered line could be assigned as either a tumor or a background pixel. An intensity profile of the line was plotted. In addition to the intensity profile, a TBR of the line (each individual pixel intensity divided by the mean of all background pixel intensities of the respective line), and an aCNR of the line (using each individual pixel intensity, the MFI of the background pixels and the standard deviation of the background pixels [Eq. (3)] was plotted. Finally, a mean TBR and aCNR were calculated for the line.

Interestingly, we found that based on the aCNR a tumor boundary could be detected. Because both tumor and background tissue encompass noise, the position at which a signal rises above twice the standard deviation of the background, one would expect the tumor boundary. Based on the aCNR plot, an area under the curve (AUC) of the tumor pixels was also calculated.

Finally, on a per-pixel basis, an R-squared and bias [standard deviation (SD) of bias] were calculated using simple linear logistics and a Bland-Altman plot, respectively. Also, a distinction between tumor and background tissue pixels was made based on final histopathology to assess the discriminating ability of each system. Based on this, the percentage of tumor pixels below the maximum background intensity was determined.

#### 2.3.5 Statistical analysis

MFI analyses were performed using ImageJ. Data was tested for Gaussian distribution using Anderson–Darling and Shapiro–Wilk tests; none of the data was normally distributed. Statistical differences were tested using a Wilcoxon test, p values<0.05 were considered significant. GraphPad Prism (version 9.1.0, GraphPad Software Inc., San Diego, California, United States) was used for statistical analysis and graph design.

## 3 Results

### 3.1 Potential of IRDye800CW in the SWIR Range

**Fig. 3:**
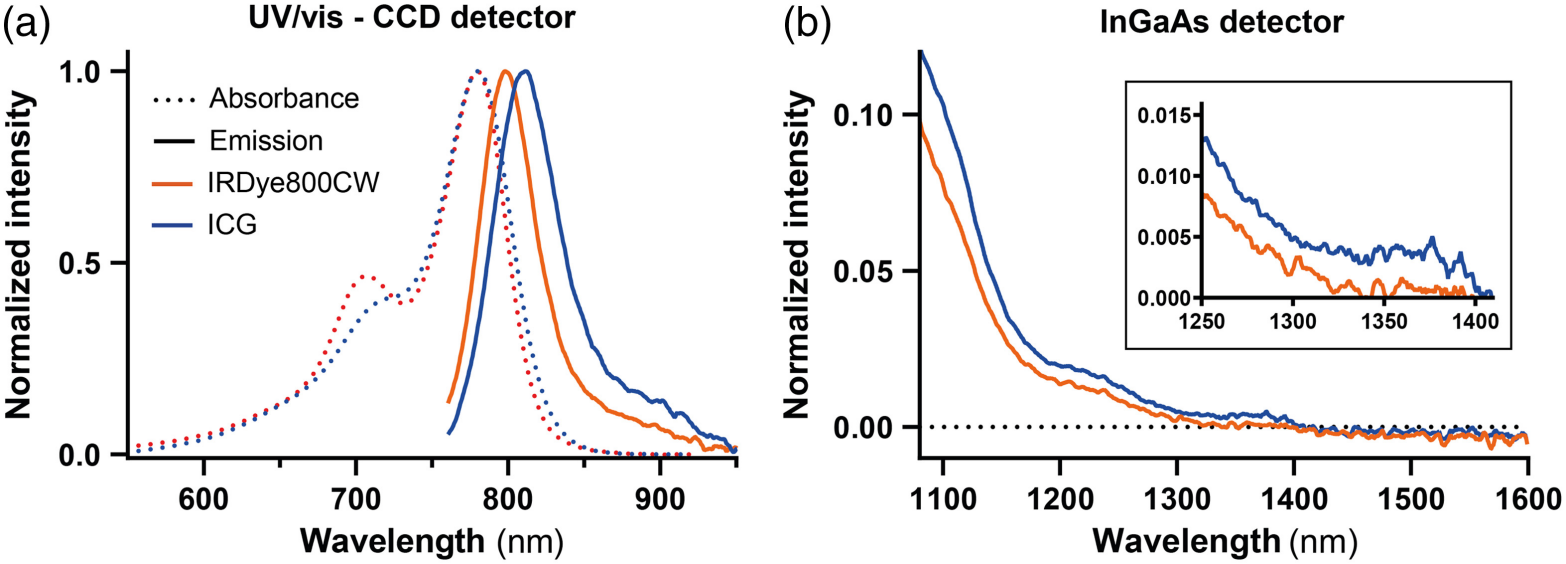
(a) Absorption spectra (dashed line) of the dyes ICG and IRDye800CW in the NIR spectral range (550 to 950 nm) and the emission spectra (solid line) of both dyes at excitation of 660 nm in the NIR spectral range (750 to 950 nm). (b) Emission spectra of the dyes at excitation of 785 nm in the SWIR spectral range (1100 to 1600 nm). The inset shows emission in the 1250 to 1400 nm range.

The absorption spectra for both IRDye800CW and ICG were recorded in the range of 500 nm up to 1000 nm [Fig. 3(a)] and showed results consistent with published literature.^45^ Subsequently, emission spectra of both dyes were recorded in the range of 750 nm up to 1600 nm (Fig. 3). ICG showed an emission maximum at 811 nm, whereas cetuximab-IRDye800CW showed an emission maximum at 798 nm. As described elsewhere,^45^ the tail emission of ICG and IRDye800CW both continue beyond 1100 nm, up to 1350 nm for IRDye800CW and >1400  nm for ICG. Therefore, detection of IRDye800CW emission is possible in the SWIR spectral region, although its intensity is not comparable to ICG, as the absolute intensity of ICG is at least three times higher. The second emission peak of ICG at around 1500 nm as reported in earlier literature,^45^ is not observed here.

### 3.2 NIR Imaging System Comparison

#### 3.2.1 Sensitivity assessment

Figure 1 summarizes the sensitivity for IRDye800CW and ICG of the imaging systems investigated for this study. Sensitivity assessment, as described in Sec. 2.2.1, showed that the PEARL Trilogy could detect concentrations down to 1 nM for IRDye800CW and ICG. The SurgVision Explorer Air II and Kaer Labs NIR-II system detected concentrations down to 10 nM for IRDye800CW and 1 nM for ICG. Quest Spectrum detected the concentrations of both dyes down to 10 nM. The detection limit of the Stryker Spy Elite is 100 nM for IRDye800CW and 10 nM for ICG. A previous study estimated the concentration of cetuximab-IRDye800CW in HNSCC to be 4.5  nmol/ml (4.5  μM), based on MDSFR/SFF spectroscopy.^2^ Thereby, the Kaer Labs NIR-II system is expected to be able to detect IRDye800CW in clinical samples.

#### 3.2.2 Solid phantom comparison

**Fig. 4:**
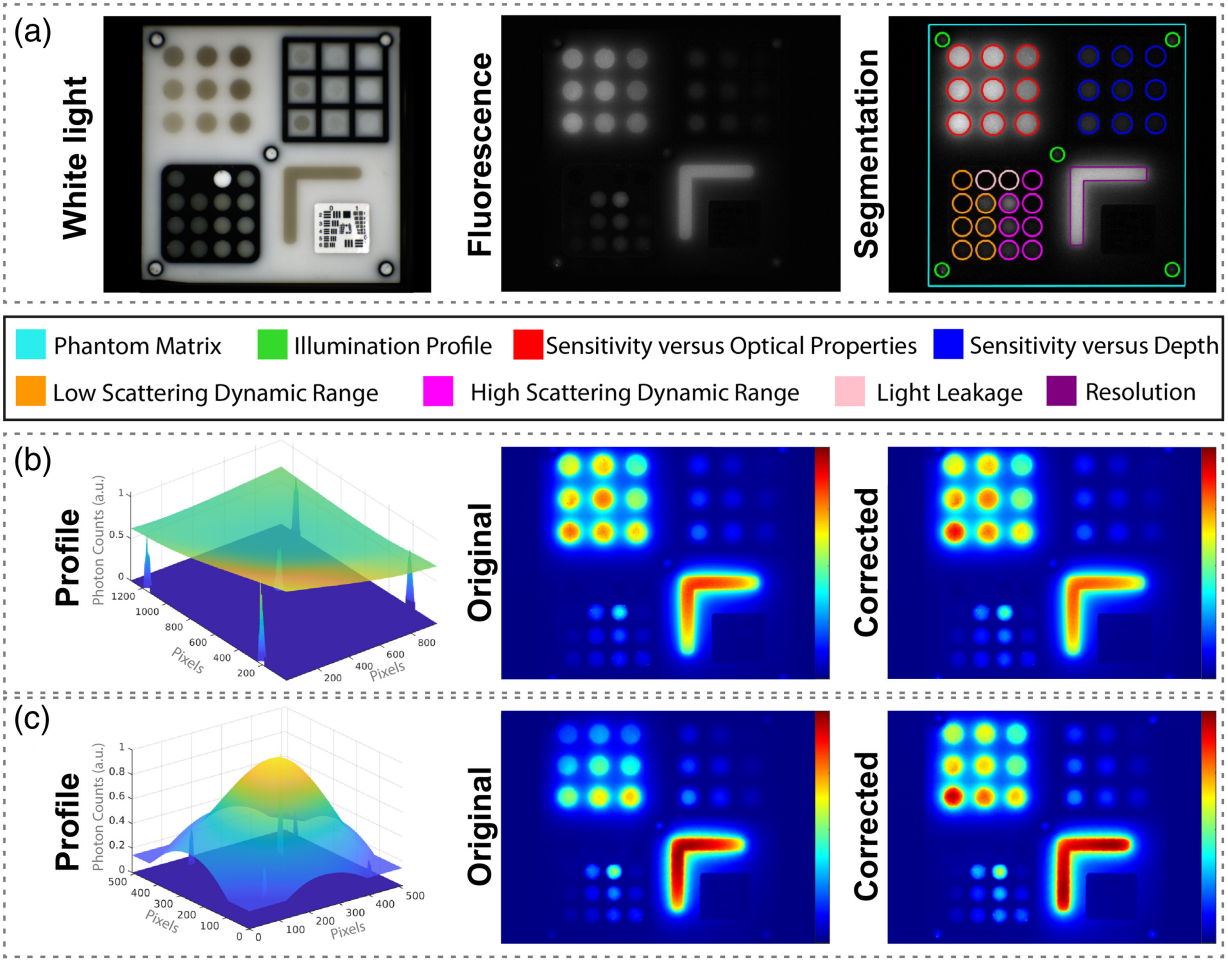
(a) Corresponding white light and fluorescence image with segmentation of various compounds for benchmarking measurements. (b)–(c) Representative examples for correction of the fluorescence images of two of the NIR fluorescence cameras: the PEARL Trilogy (b) and SurgVision Explorer Air (c) based on the signal intensity from the wells, designated to assess the illumination profile.

**Fig. 5:**
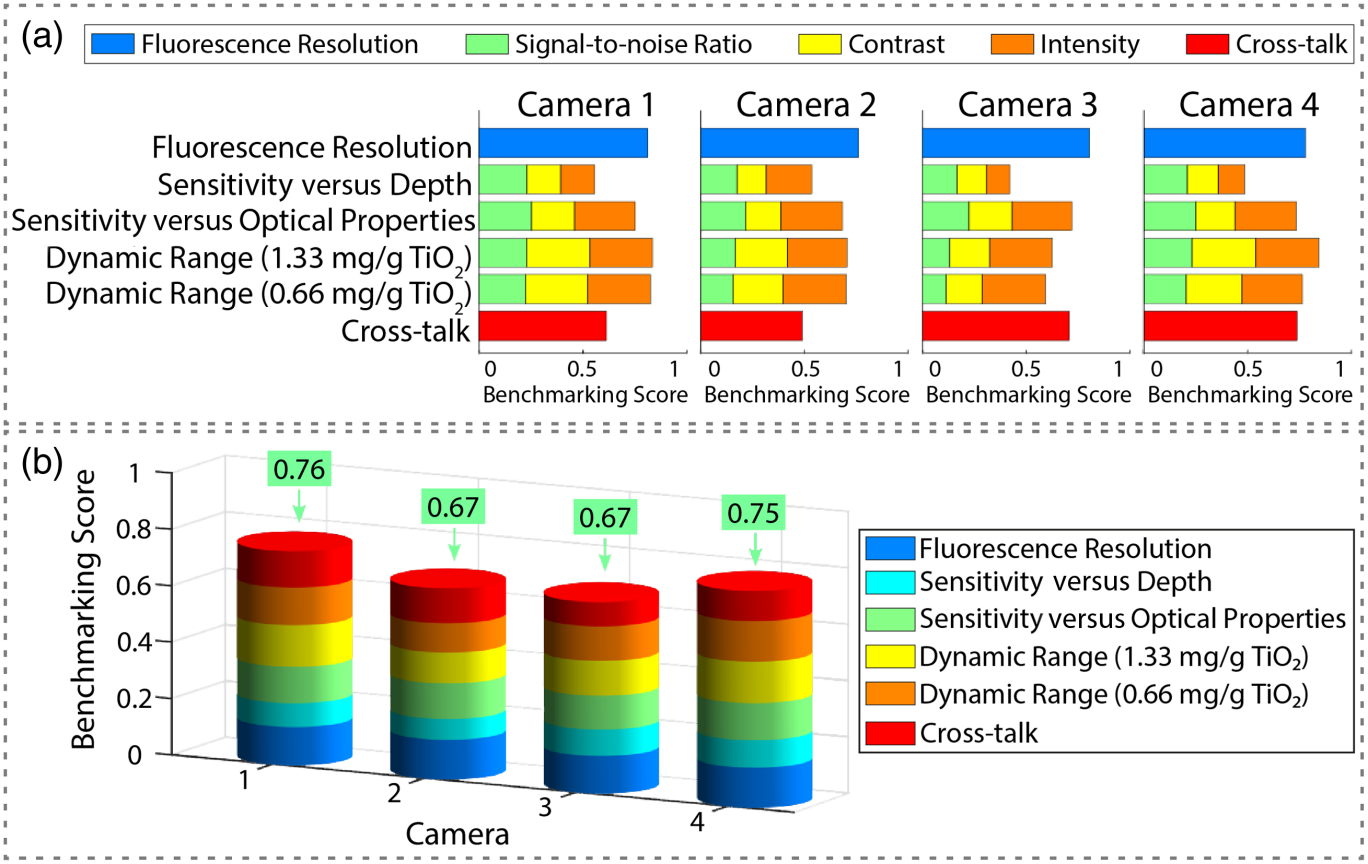
(a) Individual benchmarking scores of camera 1 (Li-COR PEARL Trilogy), camera 2 (Quest Spectrum), camera 3 (Stryker Spy Elite), and camera 4 (SurgVision Explorer Air). The individual benchmarking scores are a combination of individual metric scores. (b) The overall benchmarking scores for each camera consisting of an aggregate of individual benchmarking scores.

In line with the recent guidelines of the AAPM task group (TG311) for evaluating FMI systems,^50^ we employed the multiparametric phantom described in Sec. 2.2.2 to objectively identify the best-performing NIR system for the comparison to the SWIR camera system. Yet, as the phantom used employs quantum dots (Qdot^®^ 800 ITK™), given their stability, assessment of SWIR imaging camera performance is not possible as these quantum dots used only emit at λ=800±60  nm, i.e., in the NIR range.^52^ After segmentation and correction for variances in the illumination spatial distribution of the phantom fluorescence images [Figs. 4(a)–4(c)], benchmarking scores were calculated for all specific properties, relative to an “ideal” imaging system. Averaging these scores resulted in an overall benchmarking score, showing the best-performing imaging system [Figs. 5(a) and 5(b)].^44^ The PEARL Trilogy and SurgVision Explorer Air showed the highest benchmarking scores of 0.76 and 0.75, respectively. The Quest Spectrum and Stryker Spy Elite imaging systems showed a score of 0.67. These overall benchmarking scores confirm that the PEARL trilogy is the optimal imaging system for comparison to the Kaer Labs NIR-II system.

### 3.3 Comparison of Optimized NIR and SWIR Imaging Using Clinical Tumor Samples Containing Cetuximab-IRDye800CW

#### 3.3.1 Choosing optimal SWIR fluorescence imaging settings

**Fig. 6:**
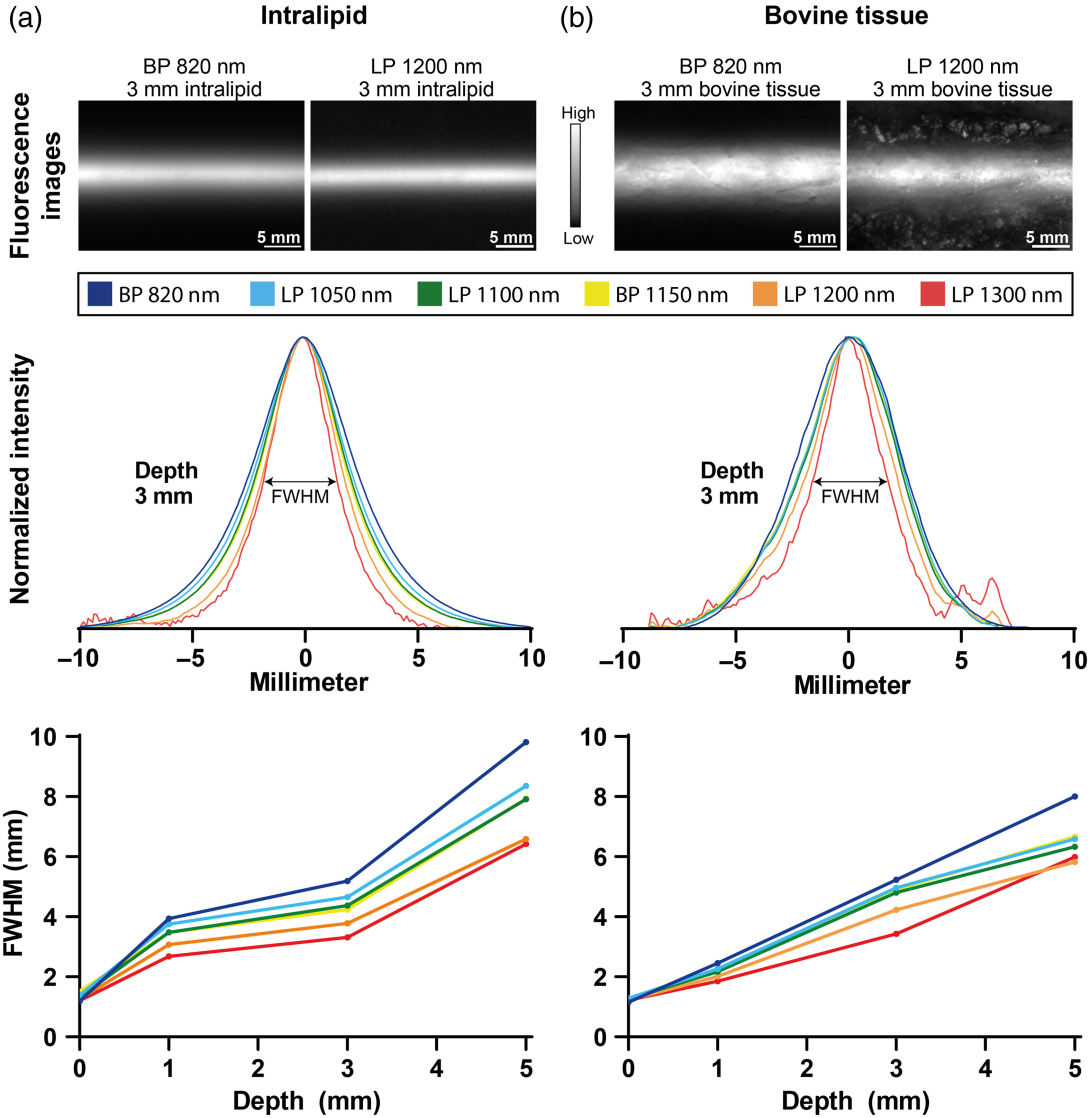
Full width at half maximum (FWHM) measurements of a small tube containing cetuximab-IRDye800CW embedded in intralipid (a) and bovine tissue (b) at depths of 0, 1, 3, and 5 mm. Intensity profiles are based on the average of normalized intensity in the fluorescence image obtained using various filters.

The optimal settings for the SWIR imaging system were chosen based on the FWHM of the fluorescence images of tubes containing IRDye800CW embedded in intralipid and bovine tissue at various depths. As shown in Fig. 6, using an LP 1300-nm filter results in the lowest FWHM for both intralipid and bovine tissue measurements. However, when inspecting the normalized intensity plot, noise becomes more apparent, possibly due to a lower maximum fluorescence intensity. The LP 1200-nm filter plot shows only a 7% average increase in FWHM while reducing contrast-inhibiting noise. Therefore, using an LP 1200-nm filter is considered optimal. Based on the results shown in Fig. 6 SWIR imaging down to a depth of 3 mm should be possible reliably.

#### 3.3.2 Comparing NIR and SWIR fluorescence images

**Fig. 7:**
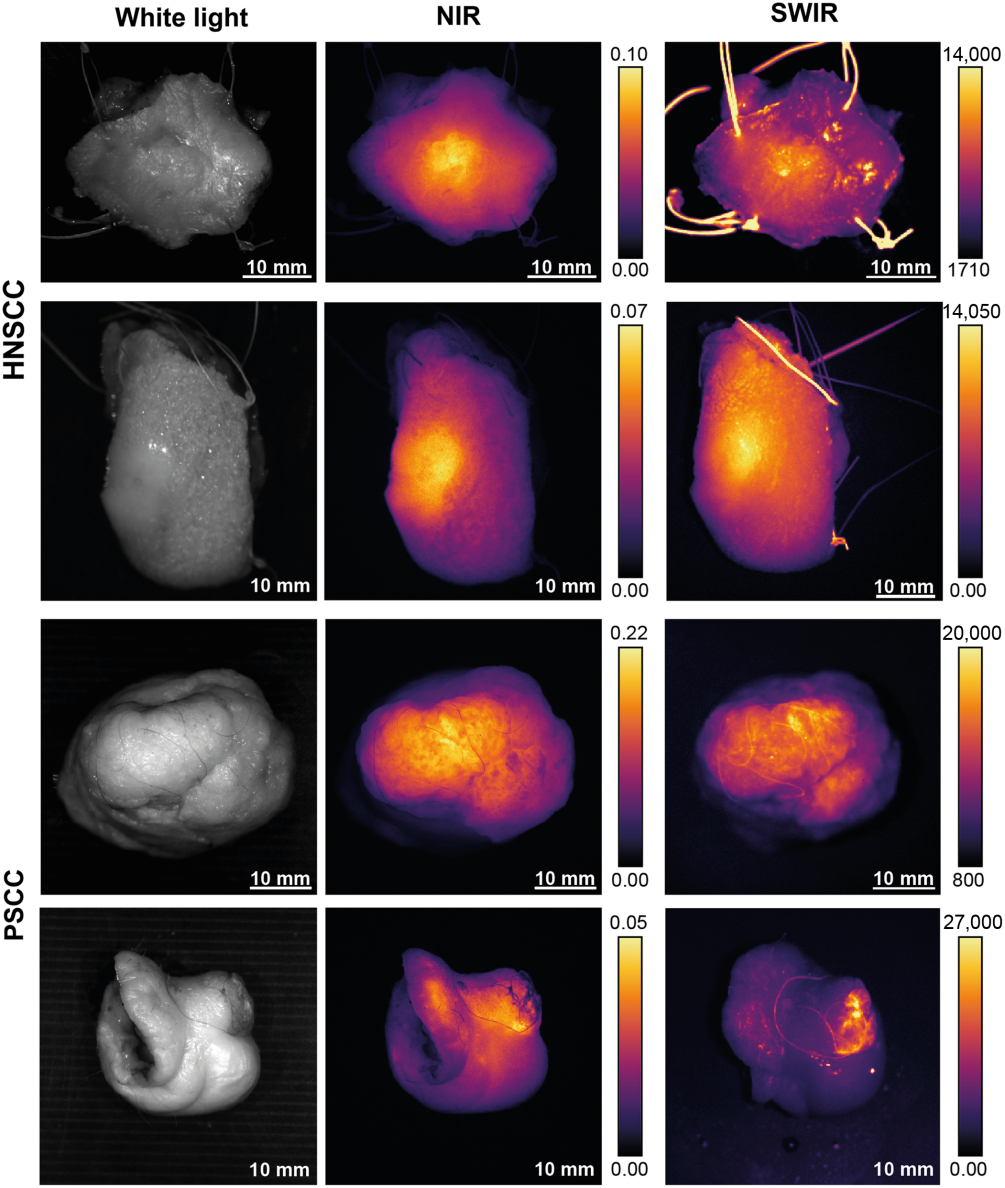
White light and NIR and SWIR fluorescence images of representative resection specimens of HNSCC (top rows) and PSCC (lower rows). The fluorescence images of the NIR and SWIR systems show similar fluorescence patterns.

A total of eight patients were included, of which six patients with PSCC and two patients with HNSCC. Fluorescence images in Fig. 7 show a similar distribution of fluorescence signals in the tumor and background. At this moment, we cannot explain the cause of artifacts on the SWIR images; however, this is discussed further in the discussion. These and other images taken suggest comparable contrast in NIR compared with SWIR images. Yet, without knowledge on the exact position of tumors from pathology analysis, objective assessment is difficult. Consequently, tissue slices were analyzed.

**Fig. 8:**
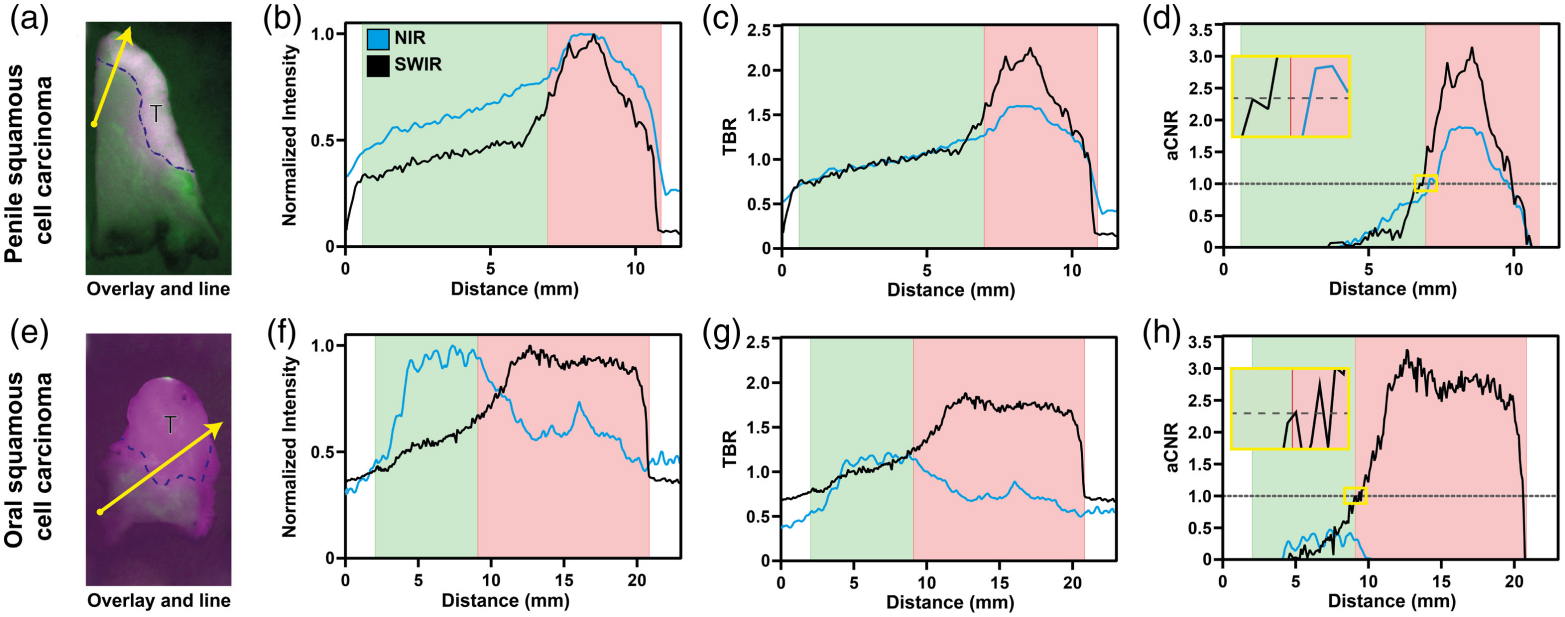
Representative examples of penile and oral squamous cell carcinoma. (a) (e) Overlays of registered NIR and SWIR fluorescence images, segmented tumor tissue (T), and line chosen for plotting values (yellow arrow). (b)–(d)/(f)–(h) Plots of normalized intensity, TBR, and aCNR, respectively, for NIR (black line) and SWIR (blue line). The red area represents tumor tissue, and the green area represents healthy tissue, as segmented by a pathologist. The yellow highlighted inset in panels d and h shows an enlargement of the aCNR = 1 crossing.

The resected specimens resulted in a total of seventeen PSCC and five HNSCC tumor-containing tissue slices, which were segmented according to pathologist assessment. For every tissue slice, four lines were drawn, resulting in 68 lines for PSCC and 20 lines for HNSCC. A number of criteria for comparing NIR with SWIR imaging for both the tissue slices and lines were evaluated. An overview of these parameters is given in Fig. 8 and Table 1.

**Table 1:** Comparison of imaging parameters between NIR and SWIR imaging systems, split into penile cancer and head and neck cancer.

Parameter	NIR	SWIR	P-value	NIR	SWIR	P value
	Penile cancer			Head and neck cancer		
TBR whole tissue slice	**1.43** (**1.15** to **1.80**)	**1.25** (**1.21** to **1.35**)	0.0359*	2.20 (1.95 to 2.61)	0.99 (0.98 to 1.03)	0.0625
aCNR whole tissue slice	0.60 (0.18 to 1.05)	0.86 (0.53 to 1.07)	0.106	1.38 (1.07 to 2.00)	–0.01 (–0.04 to 0.06)	0.0625
Median tumor fluorescence intensity (IQR) (A.U.)	0.0085 (0.021)	4189.5 (2696.5)		0.0158 (0.008)	5661 (2478)	
Median background fluorescence intensity (IQR) (A.U.)	0.0059 (0.013)	3331.3 (1937.8)		0.0073 (0.005)	5635.8 (2602.5)	
TBR over line	1.24 (1.07 to 1.47)	1.28 (1.17 to 1.38)	0.92	**1.47** (**1.34** to **1.57**)	**1.02** (**0.79** to **1.11**)	<0.0001*
aCNR over line	**0.63** (**0.10** to **1.09**)	**1.06** (**0.60** to **1.45**)	<0.0001*	**1.42** (**1.08** to **2.04**)	**0.06** (**–0.22** to **0.27**)	<0.0001*
AUC aCNR	**2.56** (**1.34** to **6.85**)	**4.81** (**2.30** to **8.48**)	0.0003*	**17.81** (**2.79** to **27.4**)	**1.09** (**0.24** to **4.10**)	<0.0001*
Absolute distance tumor border to aCNR=1 (mm)	**0.33** (**0.10** to **0.62**)	**0.53** (**0.16** to **0.87**)	0.0076*	**0.19** (**0.06** to **0.41**)	**1.02** (**0.57** to **1.80**)	0.0043*
Overlap pixels tumor/background (%)	**62.5** (**36.9** to **92.9**)	**44.0** (**22.3** to **75.7**)	0.0001*	**32.0** (**9.25** to **49.0**)	**100** (**84.5** to **100**)	<0.0001*

Abbreviations: tumor-to-background ratio (TBR), adapted contrast-to-noise ratio (aCNR), area under the curve (AUC).

Traditionally, the TBR over a tissue slice, or as recently suggested the CNR over a tissue slice,^58^ are used as informative to assess tumor versus healthy tissue. When doing so and comparing the TBR for the whole tissue slice, a significant difference was found between NIR and SWIR imaging in PSCC tissue slices in favor of the NIR imaging. However, when assessing HNSCC, no significant difference was found, neither for the aCNR over the whole tissue slice in PSCC nor in HNSCC (see Table 1).

To compare NIR and SWIR imaging performance, assessment of the drop-off of the measured signal beyond the tumor boundary seems a useful aspect to consider. Thus, a comparison of the NIR and SWIR signal was performed over a line crossing from the tumor into the background tissue, see Fig. 8. First, we considered whether the lowest fluorescence intensity observed in the tumor would provide a threshold of the minimum fluorescence characterizing a pixel in the tumor. The number of pixels with fluorescence intensities in the background exceeding this threshold value would indicate the robustness of this criterion and can be compared between the NIR and SWIR data. This comparison results in a pixel-based scatterplot of the normalized fluorescence intensities of NIR and SWIR (see Fig. S2 in the Supplementary Material). The contrast was calculated using the overlap between the intensity of pixels in tumor tissue versus pixel intensity in background pixels, SWIR showed significantly less overlap for PSCC, whereas SWIR showed significantly more overlap in HNSCC. Simple linear regression of the pixel intensity plots showed a median $r^{2}$ of 0.550 (0.233 to 0.740) for PSCC and 0.260 (0.0625 to 0.385) for HNSCC. Bland-Altman analysis showed a bias of 0.0384 (σ=0.175) and 0.0957 (σ=0.257) for PSCC and HNSCC, respectively.

**Fig. 9:**
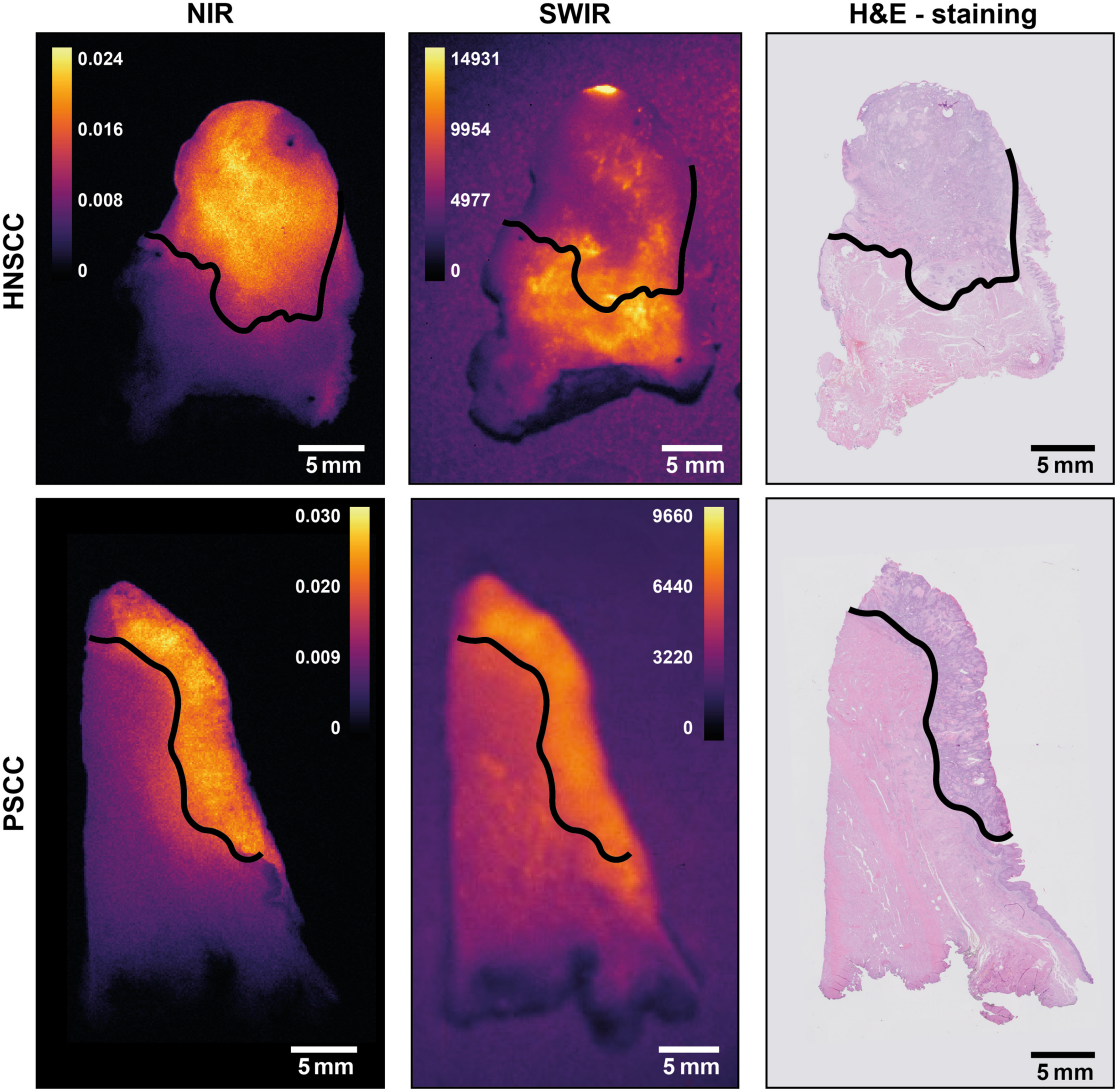
Examples of NIR and SWIR fluorescence images for oral (HNSSC) and penile (PSCC) squamous cell carcinoma compared with a hematoxylin and eosin (H&E) staining. The black line represents the tumor boundary, above this line in each image is the tumor, and below is the healthy tissue. Note that for PSCC, NIR and SWIR images indicate the same area, whereas for HNSSC, this is not the case (see text).

Finally, three different metrics were considered. First, plotting the normalized intensity of pixels over a line [Fig. 8(b/f)] showed comparable graphs for the NIR and SWIR in PSCC, whereas HNSCC showed a different intensity distribution in the comparison. Subsequently, TBR and aCNR over the corresponding lines, using pathology segmentation, were calculated and plotted [Figs. 8(c/g) and 8(d/h), respectively]. The mean TBR over a line showed no significant difference between NIR and SWIR for PSCC, but a significant difference in favor of NIR was found for HNSCC. When comparing the aCNR over a line, SWIR outperforms NIR significantly for PSCC, whereas in HNSCC, NIR outperformed SWIR significantly (see Table 1). Identical results were found when comparing the AUC of the aCNR curve. When inspecting the HNSCC sample in more detail (Fig. 9), the NIR signal is found to correspond to tumor tissue, whereas the SWIR signal does not. The role of background autofluorescence in the SWIR HNSCC images became apparent when evaluating the mean number of counts in the tumor and background in the SWIR images of PSCC and HNSCC. For tumor tissue, these are comparable, yet the number of counts in the background of HNSCC samples is higher compared with PSCC (see Table 1). In this situation, background autofluorescence in the SWIR spectral range compared with none in the NIR spectral range was also observed in the tissue of HNSCC patients without any tracer (Fig. S1 in the Supplementary Material). This result seems to match the overwhelming tissue autofluorescence in the whole SWIR region as found in a mouse study by Diao et al.^36^

Interestingly, while comparing the NIR and SWIR images based on aCNR, we observed that the aCNR curve appears to cross at aCNR = 1 at the tumor border as designated by pathology analysis, whereas the absolute intensity and TBR appear to have no relation to the tumor border [Figs. 8(d/h) versus 8(b/f) and 8(c/g), respectively]. Therefore, the absolute distance of the aCNR = 1 crossing to the tumor border based on pathology was calculated. For PSCC, a positive agreement for finding a crossing was 91.8% and a negative agreement of 36.8% was found. For HNSCC, a positive agreement of 75.0% was found. A negative agreement could not be determined because NIR found a crossing in all cases. The median distance between the aCNR = 1 crossing and the crossing to the real tumor border was significantly lower for NIR in both PSCC and HNSCC.

## 4 Discussion

In surgical oncology, complete removal of tumor tissue is the first and most essential element in the treatment of cancer patients. Postoperative TPM necessitates additional treatments (i.e., re-operation, chemo- and radiotherapy), increasing the risk of complications and patient morbidity. Owing to its ability to provide real-time intraoperative visualization of tumor tissue, fluorescence imaging is investigated. Thus far, predominantly, NIR fluorescence imaging has been used. However, SWIR fluorescence imaging recently has gained interest because of the improved availability of SWIR detectors. Furthermore, the expected optical properties of the tissue in the SWIR region (i.e., lower autofluorescence, reduced scattering, and a higher tissue penetration depth^35^^–^^37^) may lead to improved contrast over NIR imaging. A recent study, investigating multispectral NIR and SWIR imaging using ICG in liver tumors, showed higher tumor–detection sensitivity and tumor-to-normal-liver-tissue ratio for SWIR fluorescence imaging compared with NIR imaging.^59^ Two recent animal studies comparing NIR and SWIR imaging using ICG and IRDye800CW in mice also showed an improvement in contrast to SWIR imaging using the emission tail of these dyes.^45^^,^^46^^,^^60^ The use of SWIR emission tails of NIR dyes, of which some are already FDA-approved, is attractive as it might lead to a swift translation of SWIR imaging to the clinical setting.

Here, the potential of SWIR fluorescence imaging in human clinical samples using cetuximab-IRDye800CW was investigated. Emission spectra of ICG and IRDye800CW were collected and confirmed the potential of SWIR imaging of both dyes (Fig. 3). The emission signal of IRDye800CW extends up to 1350 nm, while the emission of ICG extends up to 1400 nm. A second emission peak for ICG in the SWIR spectral range between 1500 and 1600 nm, reported in earlier literature, was not observed.^45^ An explanation for this may be that in earlier measurements, the detector picked up the second-order diffraction of the excitation laser. Whether adequate detection of an emission signal up to 1350 nm for IRDye800CW in human tissue can be obtained depends on the imaging setup (i.e., imaging system, surrounding light, angles of illumination and detection, and tissue properties).

For the NIR-SWIR comparison, the key NIR imaging systems used clinically were compared. Based on a comparison using both liquid phantoms with the NIR dyes of interest (Fig. 1) and an independent solid phantom comparison (Fig. 5), the PEARL Trilogy system was determined to be the best NIR system for comparison to the SWIR system. For this comparison, as the PEARL Trilogy is a closed-field imaging system, the SWIR system was placed in a purposely built light-tight box. The imaging systems compared in this study are a dedicated and fully developed NIR system and a first-generation SWIR system with adequate sensitivity for fluorescent NIR dyes (Fig. 1). Further development in automation and SWIR detector technology may occur, and the results must be interpreted in that light.

Although real-time fluorescence-guided surgery is performed on tissue resection specimens, a meaningful comparison between both imaging modalities requires a ground truth through the golden standard: H&E pathology assessment. Therefore, the comparison was performed on tumor-containing tissue slices, on which a board-registered pathologist had indicated the tumor border. In addition, the use of tissue slices with a smooth surface allows for the acquisition of comparable fluorescence images for both systems, which in turn facilitates better image registration (Fig. 2). Finally, using tissue slices might reduce the positive effects encountered with SWIR imaging in terms of deeper tissue penetration and therefore reduction of scattering. SWIR shows its advantage best when the sample thickness is greater than the penetration depth of NIR imaging because in that case, SWIR images a larger volume. In this study, tissue slices of 4- to 5-mm thick are used and tissue penetration of NIR imaging is estimated to be ∼2.2  mm.^39^ Therefore, in the slices, SWIR still is expected to show an advantage over NIR in terms of penetration depth.

The quantitative comparison of the fluorescence images of the whole tissue slices for both NIR and SWIR imaging only showed a significantly higher TBR for NIR imaging in PSCC. This would indicate that, given whole tissue slices, in NIR a higher fluorescence intensity in the tumor compared with background tissue is detected. However, because aCNR showed no difference, the higher TBR is probably caused by a larger spread in pixel intensities, increasing the σ and thus decreasing aCNR [see Eq. (3)]. Although a difference between NIR and SWIR for TBR and aCNR was found for HNSCC, the differences were not significant.

Yet, in surgical oncology, tumor margin contrast and differences in pixel intensity over the tumor border are potentially more relevant. Using lines perpendicular to the tumor border [Fig. 8(a/e)], contrast and pixel intensities of both systems were compared. In PSCC, the aCNR over a line and the AUC of aCNR in the tumor are significantly higher for SWIR imaging, and the TBR shows no difference. This, again, indicates the influence that a higher fluorescence variation σ in the background has on NIR images. Nevertheless, NIR showed a smaller absolute distance to a tumor border. Although significant, the observed difference in median distance was only 0.2 mm, which might be caused by either a manual segmentation error and/or by the semi-automatic image registration in combination with respective resolutions. The clinical relevance of this deviation is questionable as a surgical knife has a thickness of 0.1 to 0.5 mm. For HNSCC, NIR imaging performance is significantly better than SWIR imaging in all aspects. This is caused by a discrepancy between SWIR fluorescence pattern and pathology results, whereas NIR showed fluorescence patterns corresponding to pathology results (Fig. 9). As data of patient tissue without tracer also shows (see Fig. S1 in the Supplementary Material), some components of tongue tissue appear autofluorescent in the SWIR region, thereby overwhelming the targeted fluorescence signal. This is consistent with what is reported in a mouse study by Diao et al.^36^ These authors found that only when imaging in the NIR-IIb (1500 to 1700 nm) region autofluorescence was suppressed, whereas imaging using the whole SWIR region or just the NIR-IIa (1300 to 1400 nm) region autofluorescence overwhelmed the fluorescence signal of the SWNT (single-walled carbon nanotubes) fluorophore used. In line with these results (autofluorescence in the mouse intestine and liver), we expect the autofluorescence to be due to blood and muscle content such as water at around 1300 to 1400 nm and porphyrin compounds, as the tongue tissue is highly muscular and well perfused.^61^^,^^62^ Note that the clinically approved dyes have no emission above 1400 nm [see Fig. 3(b)]. Hence, in clinical samples using a targeted NIR tracer for off-peak SWIR-imaging does not provide any benefit in line with previous results.^36^ Thus, the autofluorescence phenomenon should be considered in future research on off-peak NIR dye SWIR imaging, as it might limit SWIR fluorescence imaging based on this approach, at least in certain tissue types or applications.

Finally, the pixel-based scatterplot (Fig. S2 in the Supplementary Material) of normalized NIR and SWIR intensities was assessed. Again, SWIR outperformed NIR for PSCC indicating a higher discriminating ability between tumor and background tissue. This is presumably caused by the higher σ in NIR images. Similar to previous results, NIR showed a significantly better discriminative ability for HNSCC, as simple linear regression showed a low $r^{2}$ of 0.550 for PSCC and a low $r^{2}$ of 0.260 for HNSCC. Furthermore, based on the bias and standard deviation of the Bland-Altman plot, SWIR images could deviate from NIR images up to almost 35% and 51% (2×σ) for PSCC and HNSCC, respectively. This further demonstrates a disagreement between NIR and SWIR measurements, regardless of tissue type.

In summary, the results in Table 1 indicate that depending on tissue type, the performance of fluorescence imaging systems differs. For PSCC, NIR is better than SWIR imaging based on the current standard TBR. However, the line-based parameters for PSCC show the potential of SWIR being used in clinical tumor samples, in contrast to HNSCC. For HNSCC, NIR showed significantly better results on all fronts.

The increase in the contrast reported in previous animal studies investigating SWIR fluorescence imaging,^45^^,^^46^ has been shown in preclinical measurements here as well (Fig. 6). Although the benefit is not as apparent in clinical samples, a possible explanation for this is the difference in concentration of the tracer used. Both the ICG tracer in previous studies and the cetuximab-IRDye800CW tracer in this study were administered at a dose of $∼0.2\;\mathrm{mg}/{\mathrm{kg}}^{2}$. However, given the difference in molar mass between both tracers, the administered ICG fluorophore concentration is 100× higher than cetuximab-IRDye800CW. Increasing the cetuximab-IRDye800CW dose by 10 to 20 mg has been shown not to yield higher fluorescence intensities or higher TBR.^2^ Increase to a higher dose might lead to toxicity and side effects and is therefore undesirable. Thus, for investigating the potential role of SWIR imaging in the clinical setting, the optimal administration protocol has been used. However, using dedicated SWIR dyes^43^ rather than emission tails of NIR dyes might change the SWIR imaging performance that can be achieved. Yet, this requires regulatory approval for the clinical use of such SWIR dyes. Possibly, these dyes could overcome the background autofluorescence observed in SWIR imaging in well-perfused tissue samples.

We note that an aCNR plot over a line, perpendicular to the tumor boundary according to H&E staining, appears to be a highly relevant parameter for the comparison of tumor boundary. Using the aCNR as defined in Eq. (3), the aCNR=1 line crosses the aCNR plot at the tumor boundary in accordance with pathology assessment with a deviation within three times the resolution of the imaging system for both NIR and SWIR imaging systems in PSCC (see Fig. 8 and Table 1). The findings in HNSCC images showed similar results for the NIR system while showing higher deviations for the SWIR imaging system. Note the aCNR used here differs from the definition in earlier literature,^58^ in terms of standard deviation [two σ instead of one, see Eq. (3)] and pertaining to a line rather than an area. The results reported on the use of the aCNR criterion to determine the tumor boundary, consistent with pathology assessment, calls for further investigation in other tumors using other tracers. Such a study is currently underway.^63^

## 5 Conclusion

In a first comparison using clinical tissue samples containing a targeted dye, we show that off-peak SWIR fluorescence imaging using the targeted tracer cetuximab-IRDye800CW currently provides no additional benefit compared with NIR imaging. For this an important reason might be that the administered tracer dose in clinical studies is a hundred times lower than that used in preclinical studies. Future dosing studies should investigate this. A further aspect to consider is that in some tissue types, background fluorescence overwhelms the off-peak fluorescence of NIR tracers in the SWIR spectral range, which limits this approach to SWIR imaging. The use of new dedicated SWIR dyes, when approved for clinical use, may change this. In addition, we developed a method to perform a systematic comparison of fluorescence images, using an adapted contrast-to-noise ratio that identifies fluorescence borders and appears to work for different fluorescence systems, operating at different wavelengths, consistent with pathology analysis.

## Supplementary Material



## Data Availability

The datasets generated during and/or analyzed during the current study are available from the corresponding author on reasonable request.
