# Supplementary material for: Systematic comparison of fluorescence imaging in the near-infrared and shortwave-infrared spectral range using clinical tumor samples containing cetuximab-IRDye800CW
Source: J Biomed Opt. 2024 Nov 15;30(Suppl 1):S13708. doi: 10.1117/1.JBO.30.S1.S13708 (PMC11566260; doi:10.1117/1.JBO.30.S1.S13708)
Supplement: Supplementary file 1 [file JBO_030_S13708_SD001.pdf]

Appendix A: Supplemental Material

Figure S1 shows the NIR and SWIR images of the tissue slices of a patient with head and neck cancer with and without tracer. The figure shows that for the control patient (without tracer), a clear SWIR signal is visible in all tissue slices. However, for the NIR images barely any signal is detected. When comparing this to the patient who did receive a tracer, for SWIR similar fluorescence signals are observed. However, for NIR clearly a signal is present in the tissue slices with tracer compared to no signal for the patient without tracer.

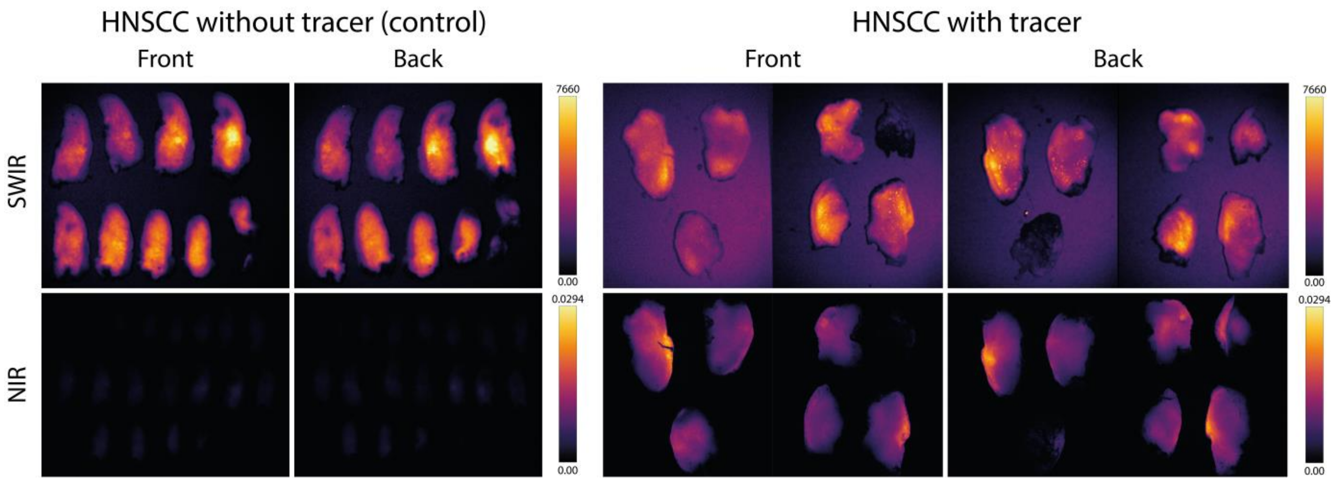

Fig. S1 Overview of tissue slices of patients with head and neck squamous cell carcinoma (HNSCC). On the left, SWIR and NIR fluorescence images of tissue slices of a patient without any tracer are shown (control) and on the right the tissue slices of a patient with a tracer. NIR and SWIR images are scaled the same between patients.

Figure S2 gives an overview of the normalized intensity, tumor-to-background ratio (TBR) and adapted contrast-to-noise ratio (aCNR) plots, similar to Fig. 7 of the manuscript. In the bottom part of Fig. S1, a scatterplot is given of the normalized fluorescence intensity of the SWIR signal versus the NIR signal over the lines shown in the top of Fig. S1. The fluorescence intensities are normalized to the highest intensity observed for NIR and SWIR, respectively. For the penile cancer sample (left column) both data lines correlated well ($r^2 = 0.939$), while for the head and neck cancer sample (right column) there is no correlation ($r^2 = 0.181$). In the latter case background fluorescence overwhelms the off-peak SWIR fluorescence signal. For both NIR (x-axis) and SWIR (y-axis), a line indicating the maximum background intensity value is drawn, giving the grey area. This grey area corresponds to the area where both NIR and SWIR indicate background intensities. All tumor values (red points) below this line indicate tumor pixels misclassified as background, when using the maximum background intensity as a cut-off. For penile cancer, a similar discriminating power can be observed for NIR and SWIR, whereas NIR outperforms SWIR for head and neck cancer.

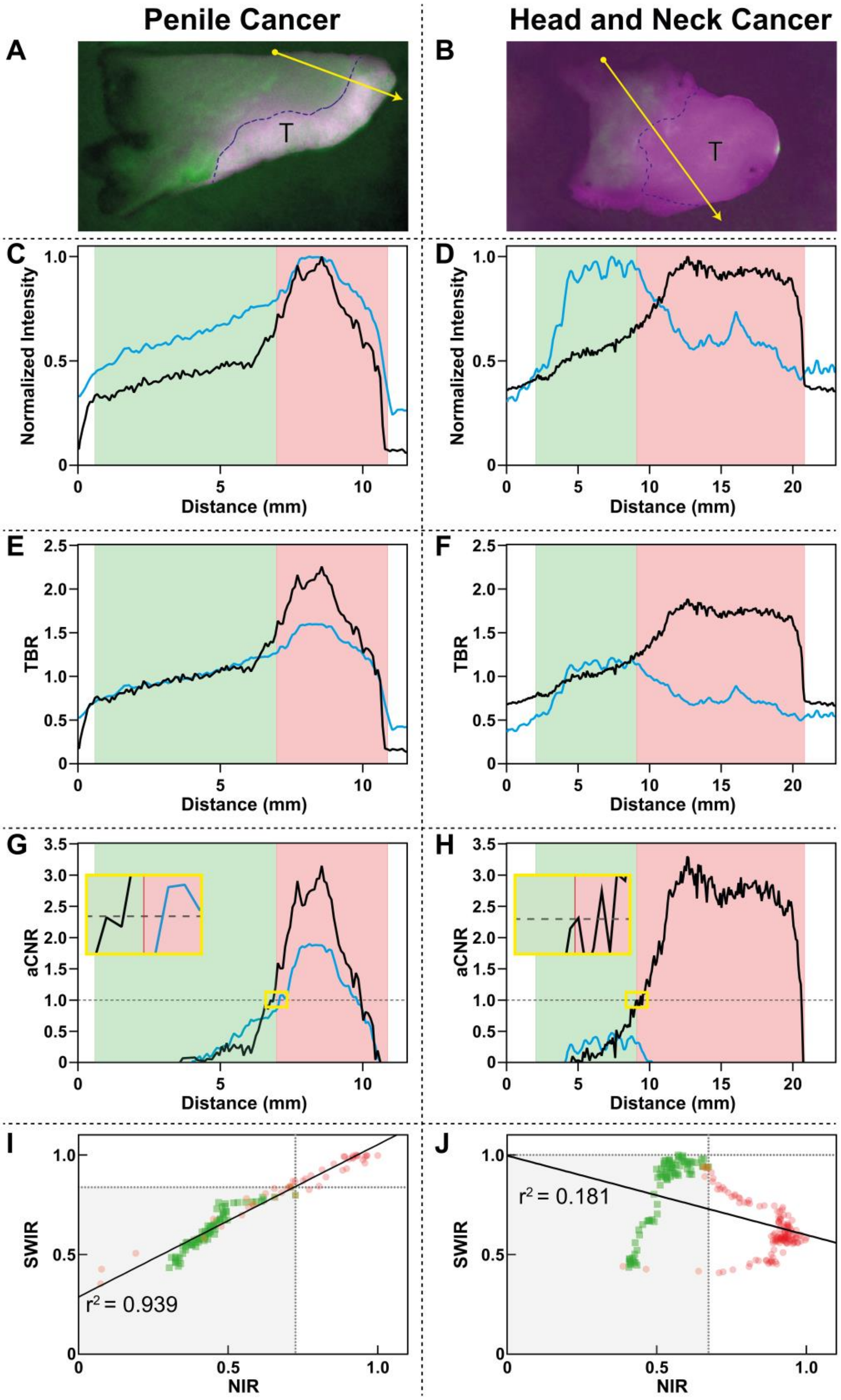

Fig. S2 Overview of the normalized intensity, tumor-to-background ratio (TBR) and adapted contrast-to-noise ratio (aCNR) plots, similar to Fig.7 of the manuscript. Ordered in columns for ease of comparison. Additionally, scatterplots of the near infrared (NIR) and short wave infrared (SWIR) normalized fluorescence intensities have been added (bottom row). The green pixels indicate background pixels as classified by a pathologist, the red pixels are classified as tumor.
